# Development and Validation of a Method to Determine CYP4V2 Enzyme Activity in rAAV-hCYP4V2 Gene Therapy Products Using a Bioluminescent Substrate Assay

**DOI:** 10.3390/molecules31162811

**Published:** 2026-08-12

**Authors:** Yiran Li, Wenhong Fan, Shuting Hou, Yue Ding, Xiuqing Jia, Xi Zhu, Yuemeng Yuan, Yufei Zhang, Yanrong Cao, Xi Qin, Chenggang Liang, Lan Wang

**Affiliations:** 1 National Institutes for Drug Control, Beijing 100050, China; yiranli202402@163.com (Y.L.); fanwh@nifdc.org.cn (W.F.); 17839978039@163.com (Y.D.); 18263871427@163.com (X.J.); 2 State Key Laboratory of Drug Regulatory Science, Beijing 100050, China; 3 Shanghai Vitalgen BioPharma Co., Ltd., Shanghai 201210, China; x.zhu@vitalgen.com (X.Z.); ym.yuan@vitalgen.com (Y.Y.); yf.zhang@vitalgen.com (Y.Z.); yr.cao@vitalgen.com (Y.C.)

**Keywords:** CYP4V2, bioluminescent substrate method, enzyme activity, adeno-associated virus, gene therapy, method validation

## Abstract

Bietti crystalline dystrophy like retinal degeneration (BCD) is an inherited retinal degenerative disease caused by defects in the CYP4V2 gene. Recombinant AAV-based gene replacement therapy holds promise for this disease, yet reliable enzymatic activity assays for product potency evaluation remain urgently needed. This study established and validated a method for determining the enzymatic activity of CYP4V2 based on the bioluminescent substrate Luciferin-MultiCYP.HEK293-AAVR cells were seeded at 1 × 10^4^ cells/well and transduced with rAAV-hCYP4V2 across an MOI gradient (1.17 × 10^3^–2.4 × 10^6^). After incubating at 37 °C for 72 h, medium was replaced with Opti-MEM containing 20 µM Luciferin-MultiCYP and incubated for 90 min ± 30 min. Next, cell supernatants were mixed with luciferase reagent, and bioluminescence was measured. Methodological validation results revealed that the method has good specificity, with an accuracy recovery rate of 100.94% ± 2.84% (RSD = 2.81%). Repeatability (GCV%) was 4.21%, and intermediate precision (RSD) was 6.16%. Linearity was good within the MOI range of 1.17 × 10^3^ to 2.4 × 10^6^ (R^2^ = 0.9902 ± 0.0061, n = 9). Lastly, the method was successfully applied to activity testing the products of three AAV serotypes (AAV8, AAV2/8, and AAV2). This bioluminescent method meets regulatory potency assay requirements and serves as an effective QC tool for process development, batch release, and stability evaluation of rAAV-hCYP4V2 products.

## 1. Introduction

Bietti crystalline dystrophy (BCD) is an autosomal recessive inherited retinal degenerative disease caused by biallelic mutations in the cytochrome P450 family 4 subfamily V polypeptide 2 gene (CYP4V2) [1,2]. It is characterized by the appearance of yellow or white crystalline lipid deposits in the retina, and this is accompanied by progressive atrophy of the retinal pigment epithelium, photoreceptors, and choriocapillaris [3,4]. The CYP4V2 protein consists of 525 amino acids, is mainly expressed in the epithelial cells of the retina and cornea, and is localized to the endoplasmic reticulum. Its physiological function is to catalyze ω-hydroxylation of medium- to long-chain saturated fatty acids (e.g., lauric acid and palmitic acid) and polyunsaturated fatty acids such as docosahexaenoic acid (DHA) and eicosapentaenoic acid (EPA). The dicarboxylic acid products of this process subsequently enter the mitochondrial β-oxidation pathway for degradation and metabolism [5,6,7,8]. Functional loss of CYP4V2 leads to impaired fatty acid metabolism in RPE cells, manifesting as an abnormal accumulation of polyunsaturated fatty acids and reduced levels of hydroxylated metabolic products. This disruption of lipid homeostasis increases reactive oxygen species (ROS) levels and induces mitochondrial dysfunction and cell apoptosis, ultimately resulting in irreversible damage to RPE cells [9]. Currently, no approved treatments are available for this disease, highlighting the urgency of developing new therapeutic strategies [10].

A recombinant adeno-associated virus (rAAV)-mediated gene transfer strategy targeting the CYP4V2 gene has provided new options and hope for the treatment of CYP4V2-related BCD patients [11]. rAAV hCYP4V2 is an investigational gene therapy product designed to deliver a functional CYP4V2 gene (in an AAV vector) directly to retinal cells using a single administration protocol, thereby fundamentally restoring enzymatic function and delaying or blocking disease progression [12]. To date, two clinical trials reported in 2024 (NCT06302608, NCT04722107) [11,13] have found no serious treatment-related safety events and have shown clear improvements. In 2025, an rAAV2 hCYP4V2 (NGGT001) 1/2 phase clinical trial (NCT05694598) was reported as ongoing, with the stated aim of further evaluating the therapeutic efficacy and safety of VGR R01 in BCD patients, and with the ultimate goal of offering a novel treatment approach for this challenging inherited disease [14].

According to regulatory agency guidelines for the production of gene therapy products (across various countries), potency is a critical quality attribute of AAV gene therapy products; therefore, an appropriate analytical method should be used to calibrate the potency of the gene therapy products and to assess batch-to-batch consistency [15,16]. Moreover, the assay chosen should reflect the product’s mechanism of action and be validated in release, stability, and comparability studies [17]. For rAAV hCYP4V2, a quantitative biological activity assay to measure the fatty acid hydroxylation function of the CYP4V2 enzyme is required. The CYP450 enzyme activity detection methods currently used fall into three main categories: chromatography coupled with mass spectrometry based on probe substrates; high-throughput screening based on luminescence principles; and (recently emerging) electrochemical biosensor methods [12,18,19]. Although the established fatty acid substrate LC-MS/MS method does directly reflect the ω-hydroxylation function of CYP4V2, the LC-MS/MS method has several disadvantages: it is labor-intensive; its throughput is low; and it cannot be adapted to high-throughput testing for quality control of gene therapy products. While electrochemical sensor methods have potential, they still face technical bottlenecks, including the problem of enzyme stability. In contrast, a luminescence assay based on the proluciferin probe does not require complex sample pretreatment, and it can rapidly quantify enzyme activity through bioluminescence signals. However, for activity detection of the CYP4V2 enzyme expressed by the rAAV-hCYP4V2 gene therapy product, no luminescent substrate method has yet been systematically optimized and comprehensively validated to meet the regulatory requirements for product quality control and release.

**Figure 1:**
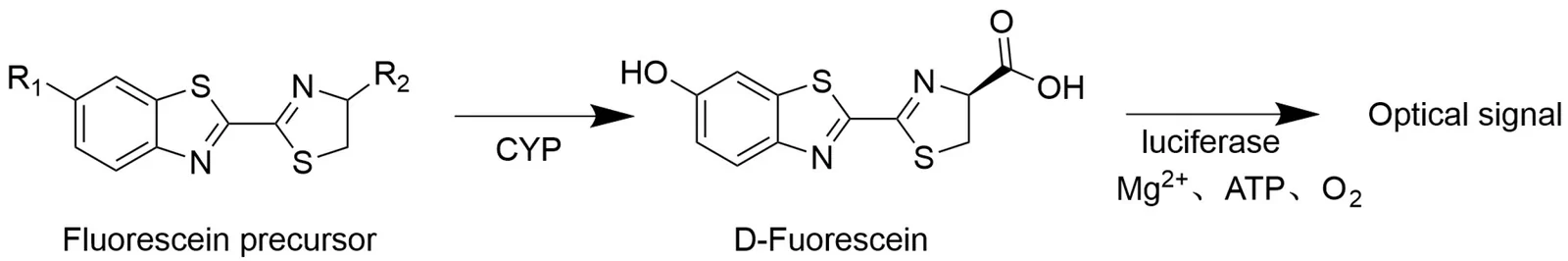
Schematic illustration of the principle of luciferase-catalyzed bioluminescence reaction.

With these issues in mind, the aim of the present study was to establish and validate a bioluminescent-substrate-based assay for detecting CYP4V2 enzymatic activity, thereby enabling direct, specific, and quantitative evaluation of the functional activity of the rAAV-hCYP4V2 product. In this study, Luciferin-MultiCYP (methyl(S)-2-(6-methoxybenzo[d]thiazol-2-yl)-4,5-dihydrothiazole-4-carboxylate) was used as a selective substrate for CYP4V2. After being catalyzed by CYP4V2, Luciferin-MultiCYP undergoes hydroxylation at a specific site, generating a fluorescent ester intermediate. This intermediate is converted into D-fluorescein under the action of the esterase contained in the fluorescent luminometer reagent. In the subsequent detection steps, the newly generated D-fluorescein is oxidized by the firefly luciferase in the presence of ATP and molecular oxygen, producing a stable and quantifiable bioluminescent signal. The intensity of this signal is directly proportional to the amount of the hydroxylation product generated, thereby directly reflecting the catalytic activity of CYP4V2 (Figure 1). Through systematic optimization and comprehensive validation, the method was designed and developed to meet the technical requirements for activity assay method validation of biological products under the general guidelines of the Pharmacopoeia of the People’s Republic of China (General Chapter 9401 “Guidance for Validation of Biological Product Bioactivity/Titer Assay Methods”), as well as other relevant international guidelines, including ICH Q2 (R2) [20,21]. In summary, the method reported here provides a reliable analytical tool for process development and quality control of the rAAV-hCYP4V2 product.

## 2. Results

### 2.1. Preliminary Confirmation of Experimental Conditions

**Figure 2:**
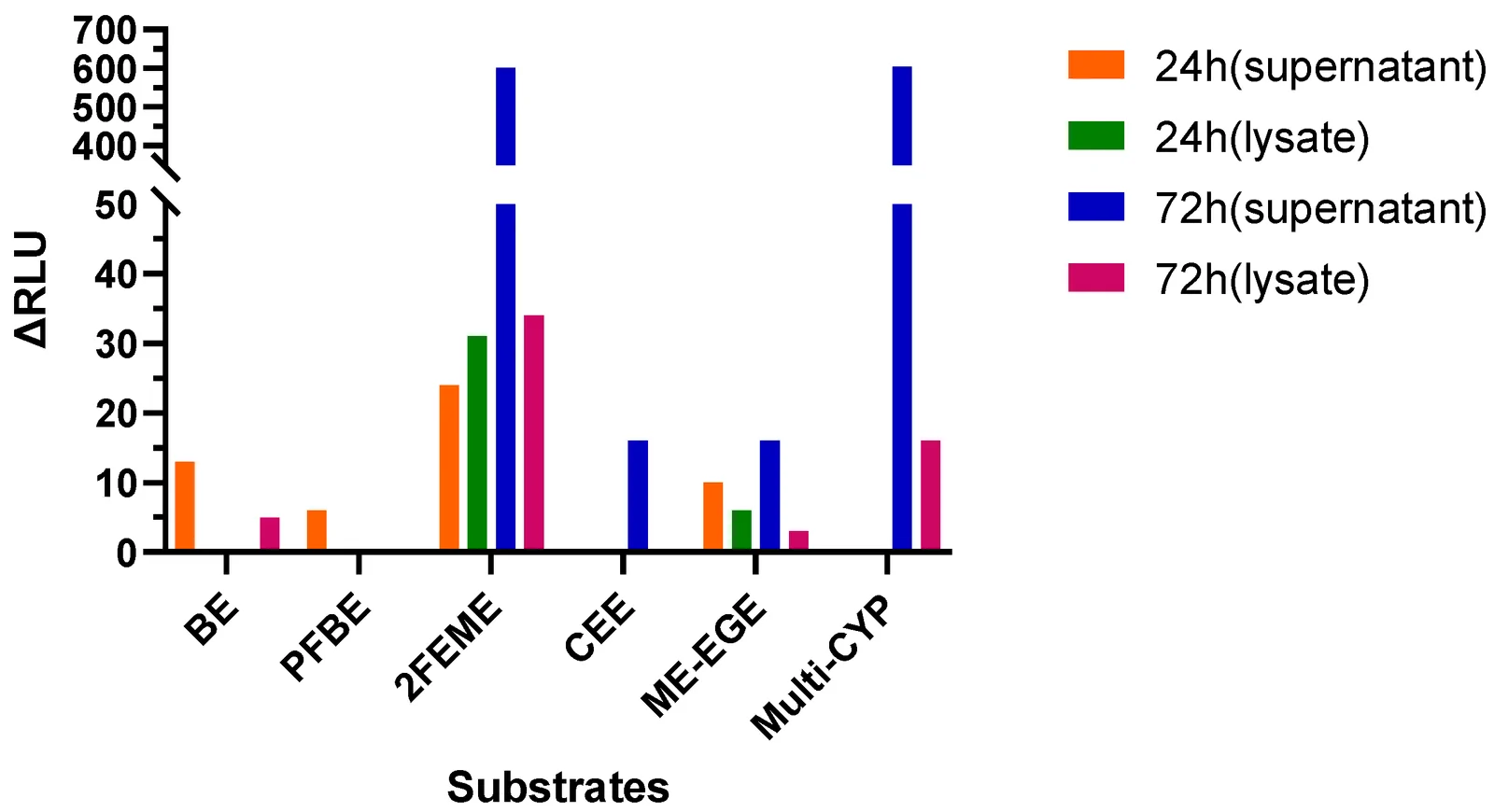
The effects of six substrates and different exchange times on the luminescent signals in the cell supernatant and the lysate.

To preliminarily determine the appropriate substrate type, exchange time, and sample type for detection (cell supernatant or cell lysate), using Hela-AAVR cells as the host cells, after being treated with HU (Hydroxyurea) (0.1 mM) for 24 h, rAAV-hCYP4V2 was transduced at an MOI of 4.8 × 10^5^. The exchange was performed 24 h or 72 h after transduction (i.e., the virus-containing supernatant was aspirated and replaced with fresh complete medium), and the cells were further cultured for 48 h before detection. The six substrates (BE, PFBE, 2FEME, CEE, ME-EGE, and Multi-CYP) were incubated with the cells at a concentration of 10 µM for 3 h. After incubation, the cell supernatants and cell lysates were collected, and the luminescence signals were measured after reacting with the D-luciferin detection reagent (mixed 1:1). The results reveal that the measurements performed on cell culture supernatants were higher than those performed on cell lysates (Figure 2, Tables S1 and S2). Moreover, of the six substrates tested, Multi-CYP and 2FEME produced better detection results than the other substrates. In addition, the results obtained after incubating with virus for 72 h and then changing the culture medium before testing were superior to those obtained after incubating with virus for 24 h and then changing the culture medium.

**Figure 3:**
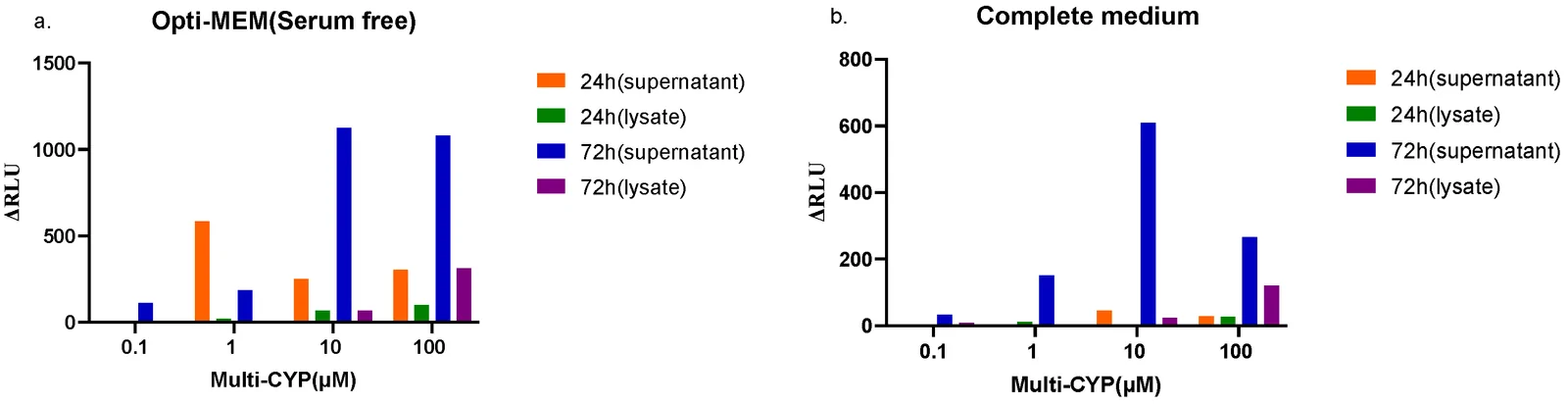
The influence of different culture media and substrate concentrations on the detection signal of Multi-CYP. (**a**) Incubation with different concentrations of substrate in Opti-MEM medium. (**b**) Incubation with different concentrations of substrate in complete medium.

Next, we assessed the optimum concentration of substrate (by varying the concentration of Multi-CYP) and also investigated the effect of culture media on signal detection. The luminescent signal values obtained using Opti-MEM medium were higher (Figure 3a); note, the associated negative control values were also higher. In comparison, incubation in complete medium resulted in lower luminescent signal values (Figure 3b); note, the associated negative control values were also lower. As shown in Tables S3 and S4, the luminescent signals were highest using substrate concentrations of 10 µM and 100 µM.

In summary, to ensure optimum viral transduction conditions, the culture medium should be replaced after 72 h of viral incubation, and the cell supernatant should be utilized as the reaction system. Additional optimization experiments facilitated identification of the optimal substrate, the optimal culture medium, and the optimal substrate concentration.

### 2.2. Method Parameter Optimization

#### 2.2.1. Cell Line Selection

**Figure 4:**
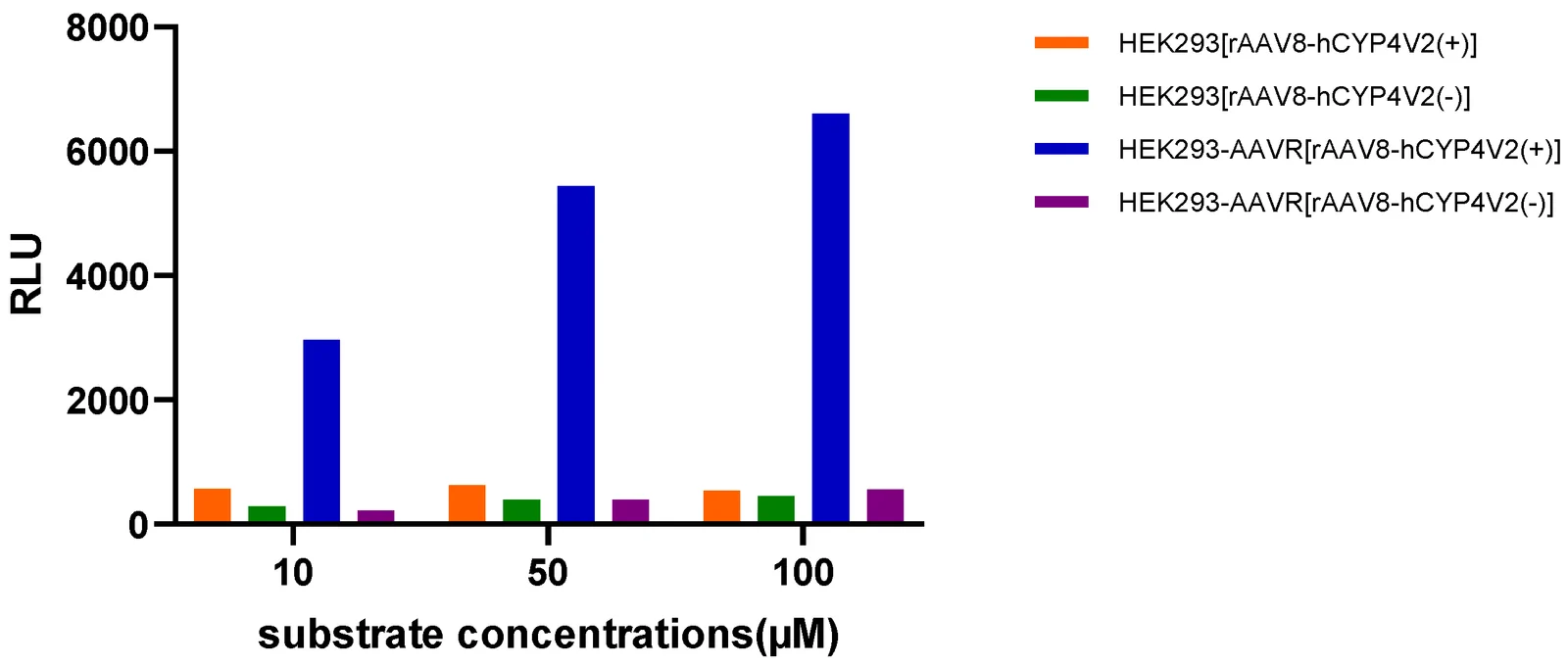
Comparison of viral transduction efficiency between HEK293 and HEK293-AAVR cell lines.

To evaluate the effects of cell line selection on viral transduction efficiency, experiments were performed using either HEK293 cells or HEK293-AAVR cells expressing the AAVR receptor. Using MOI = 4.8 × 10^5^ to transduce rAAV-hCYP4V2, the medium was changed 72 h after transduction, and the cells were further cultured for 48 h before detection. The substrate Multi-CYP was incubated with the cells at three concentrations (10, 50, and 100 µM) in the Opti-MEM medium for 3 h, and the luminescent signals in the cell supernatants were detected. Under three different substrate concentrations (10 µM, 50 µM, 100 µM), the viral transduction efficiency of HEK293-AAVR cells was observed to be higher than that of HEK293 cells (Figure 4, Table S5). Hence, the use of a cell line carrying the AAVR receptor effectively increases viral transduction efficiency, thereby improving the detection signal window. It is worth noting that the HU pre-treatment and 72 h fluid exchange followed by 48 h cultivation scheme used in the early substrate screening experiments was designed based on the low transduction efficiency of Hela-AAVR cells. After subsequent optimization by replacing with HEK293-AAVR cells with high expression of AAV receptors, the transduction efficiency significantly improved, and virus incubation for 72 h was sufficient to support the efficient expression of CYP4V2 protein. The aforementioned additional steps are no longer necessary. The final method maintains high sensitivity while significantly simplifying the operation process.

#### 2.2.2. Selection of Substrate Type and Incubation Medium

**Figure 5:**
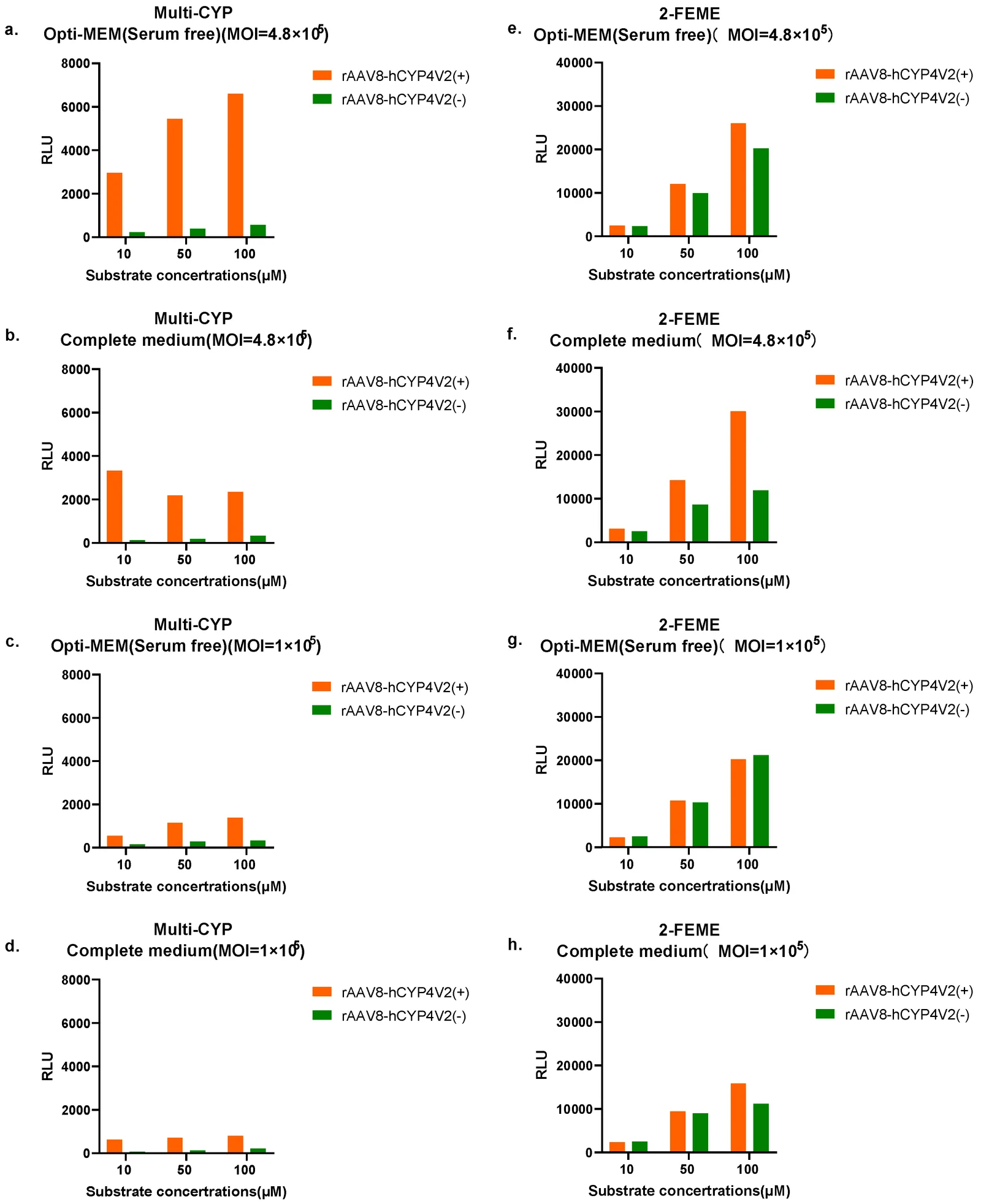
Comparison of detection windows for Multi-CYP and 2FEME substrates under different MOI, culture medium and concentration conditions. (**a**) MOI = 4.8 × 10^5^, Opti-MEM medium was used to incubate Multi-CYP; (**b**) MOI = 4.8 × 10^5^, complete medium was used to incubate Multi-CYP; (**c**) MOI = 1 × 10^5^, Opti-MEM medium was used to incubate Multi-CYP; (**d**). MOI = 1 × 10^5^, complete medium was used to incubate Multi-CYP; (**e**) MOI = 4.8 × 10^5^, Opti-MEM medium was used to incubate 2FEME; (**f**) MOI = 4.8 × 10^5^, complete medium was used to incubate 2FEME; (**g**) MOI = 1 × 10^5^, Opti-MEM medium was used to incubate 2FEME; (**h**) MOI = 1 × 10^5^, complete medium was used to incubate 2FEME.The detection window is the difference in RLU values between rAAV8-hCYP4V2(+) and rAAV8-hCYP4V2(−).

Based on the preliminary conditions determined in Section 2.1 and Section 2.2.1 (HEK293-AAVR cells, cell supernatant detected after 72 h of transduction, etc.), further evaluation was conducted on the effects of substrate type, substrate concentration, and incubation medium. At two MOI (Multiplicity of Infection) levels (4.8 × 10^5^ and 1 × 10^5^), the performance of two substrates (Multi-CYP and 2FEME) at three concentrations (10, 50, and 100 µM) in Opti-MEM or complete medium was tested, with the substrate incubation time set at 3 h. The results (Figure 5, Table S6) reveal that the detected signal values were consistently higher under the MOI = 4.8 × 10^5^ condition (when compared with the corresponding MOI = 1 × 10^5^ condition), regardless of whether Opti-MEM medium or complete culture medium was used, and for both substrates (Multi-CYP and 2FEME), regardless of substrate concentration.

Under the MOI = 4.8 × 10^5^ condition, using complete medium, the differences between rAAV8-hCYP4V2(+) and rAAV8-hCYP4V2(−) at Multi-CYP substrate concentrations of 10 µM, 50 µM, and 100 µM were 3200, 1994, and 2018, respectively. Hence, the maximum detection window under these conditions was achieved using a Multi-CYP concentration of 10 µM. In Opti-MEM medium, the differences between rAAV8-hCYP4V2(+) and rAAV8-hCYP4V2(−) at Multi-CYP substrate concentrations of 10 µM, 50 µM, and 100 µM were 2737, 5051, and 6051, respectively. Hence, the maximum detection window under these conditions was achieved using a Multi-CYP concentration of 100 µM.

Under the MOI = 4.8 × 10^5^ condition, using complete medium, the differences between rAAV8-hCYP4V2(+) and rAAV8-hCYP4V2(−) at 2FEME substrate concentrations of 10 µM, 50 µM, and 100 µM were 598, 5560 and 18168, respectively. In Opti-MEM medium, the differences between rAAV8-hCYP4V2(+) and rAAV8-hCYP4V2(−) at 2FEME substrate concentrations of 10 µM, 50 µM, and 100 µM were 123, 2092 and 5748, respectively. Hence, the maximum detection window was achieved using a 2FEME substrate concentration of 100 µM, irrespective of medium used.

In summary, under the same detection conditions, the 2FEME substrate demonstrated higher background signal values than the Multi-CYP substrate. Considering that high background signals may interfere with detection specificity, Multi-CYP was chosen for full-scale experiments as the preferred substrate.

**Figure 6:**
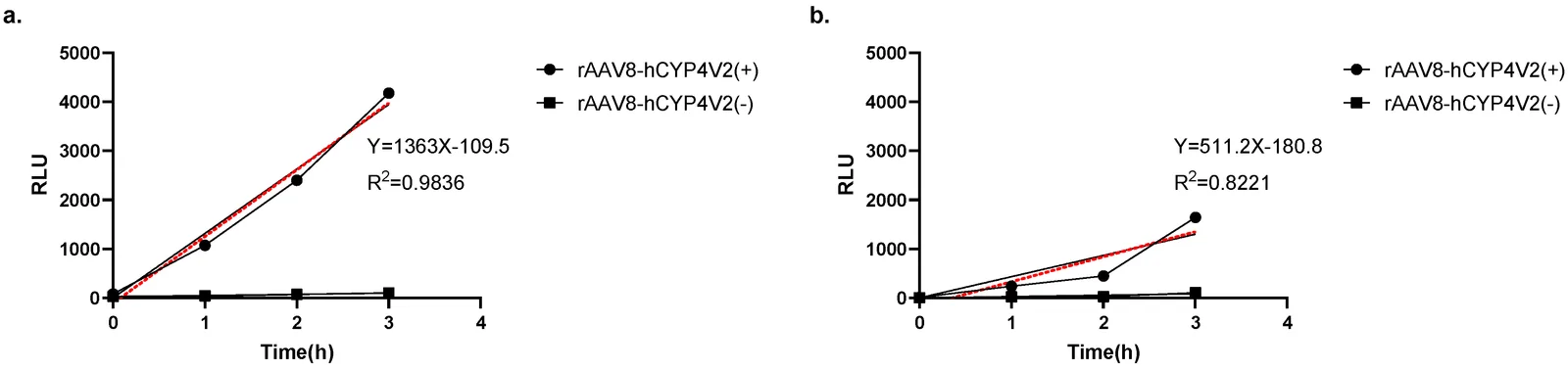
Comparison of the linear relationship between substrate incubation time and detection signal under two preferred conditions. (**a**) Results obtained using Multi-CYP (100 µM) incubated in Opti-MEM medium (for various times). (**b**) Results obtained using Multi-CYP (10 µM) incubated in complete medium (for various times). The red dashed lines represent the linear regression fits, with the corresponding equations and R^2^ values shown in the graphs.

Based on the above results, two optimal experimental conditions were selected for further comparison: 100 µM Multi-CYP, Opti-MEM medium; 10 µM Multi-CYP, complete medium. Under the 100 µM Multi-CYP/Opti-MEM conditions, a good linear relationship was observed between incubation time and the detection signal (R^2^ = 0.983). In comparison, under the 10 µM Multi-CYP/complete medium conditions, the linear relationship between incubation time and the detection signal was relatively weak (R^2^ = 0.822) (Figure 6, Table S7).

Because the 100 µM Multi-CYP/Opti-MEM medium incubation conditions yielded better results (with a good linear relationship), these conditions were chosen for subsequent experiments.

#### 2.2.3. Optimization of Substrate Incubation Time

**Figure 7:**
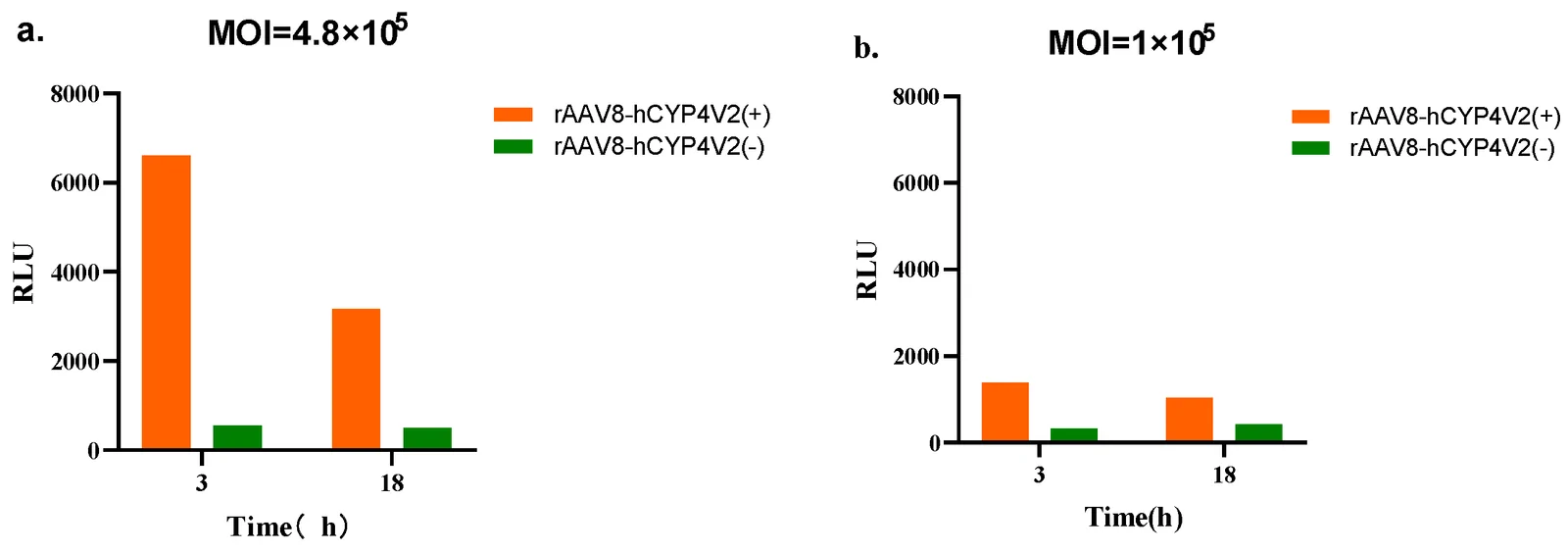
Comparison of the detection window between 3 h and 18 h substrate incubation (under both MOI conditions). (**a**) MOI = 4.8 × 10^5^, 3 h incubation vs. 18 h incubation. (**b**) MOI = 1 × 10^5^, 3 h incubation vs. 18 h incubation.

Using the substrate and culture medium conditions determined in Section 2.2.2 (Multi-CYP 100 µM, Opti-MEM medium), the incubation time of the substrate was further optimized. Firstly, the detection windows of incubation for 3 h and 18 h were compared under two MOIs (4.8 × 10^5^ and 1 × 10^5^). At MOI = 4.8 × 10^5^, the detection window was 6051 after 3 h of incubation and 2674 after 18 h of incubation. At MOI = 1 × 10^5^, the detection window was 1059 after 3 h of incubation, and 617 after 18 h of incubation. Thus, under both MOI conditions tested, the detection window obtained after 3 h of substrate incubation was greater than that obtained after 18 h of incubation (Figure 7, Table S8).

**Figure 8:**
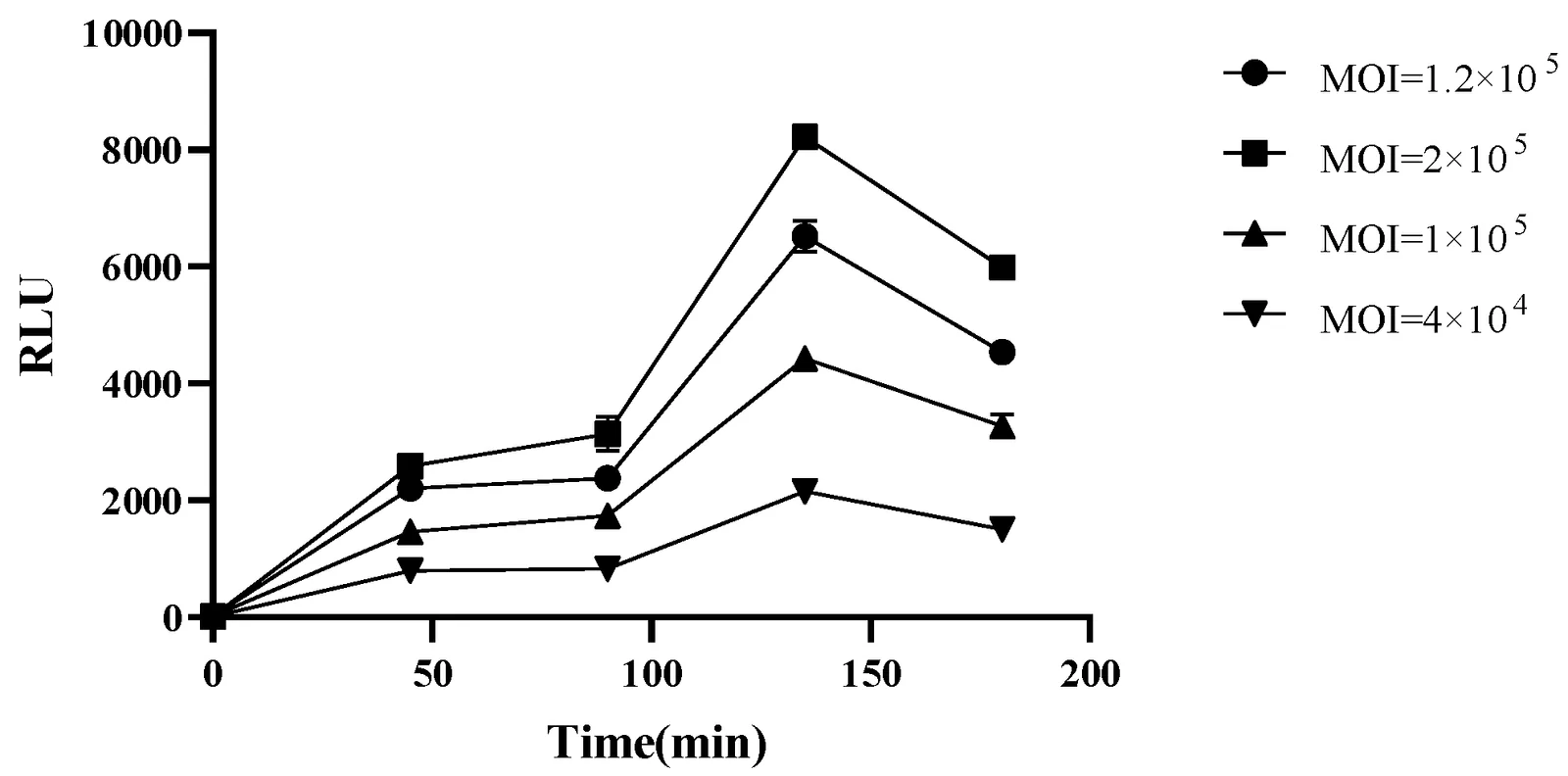
The response relationship between the incubation time of the substrate (0–180 min) and the detection signal under different MOI conditions.

Next, the response relationship between incubation time (0 min, 45 min, 90 min, 135 min, 180 min) and detection signal was assessed under four MOI conditions (2 × 10^5^, 1.2 × 10^5^, 1 × 10^5^, 5 × 10^4^) (Figure 8, Table S9). Under all MOI conditions tested, the detection signal values increased with incubation time, reaching a peak at 135 min, before dropping at 180 min. On the basis of the above experimental data, the response relationships between signal value and substrate concentration, seeded cell number, and incubation time should be further clarified to establish a four-parameter fitting calculation for EC_50_ (and thus compute relative activity). To achieve this, we optimized the MOI (with serial dilutions starting from 1.2 × 10^6^) and the fitting approach (four-parameter fitting). This allowed us to subsequently explore the optimal substrate concentration, seeded cell number, and incubation time, and thereby determine the best methodological parameters.

#### 2.2.4. Substrate Concentration Validation

**Figure 9:**
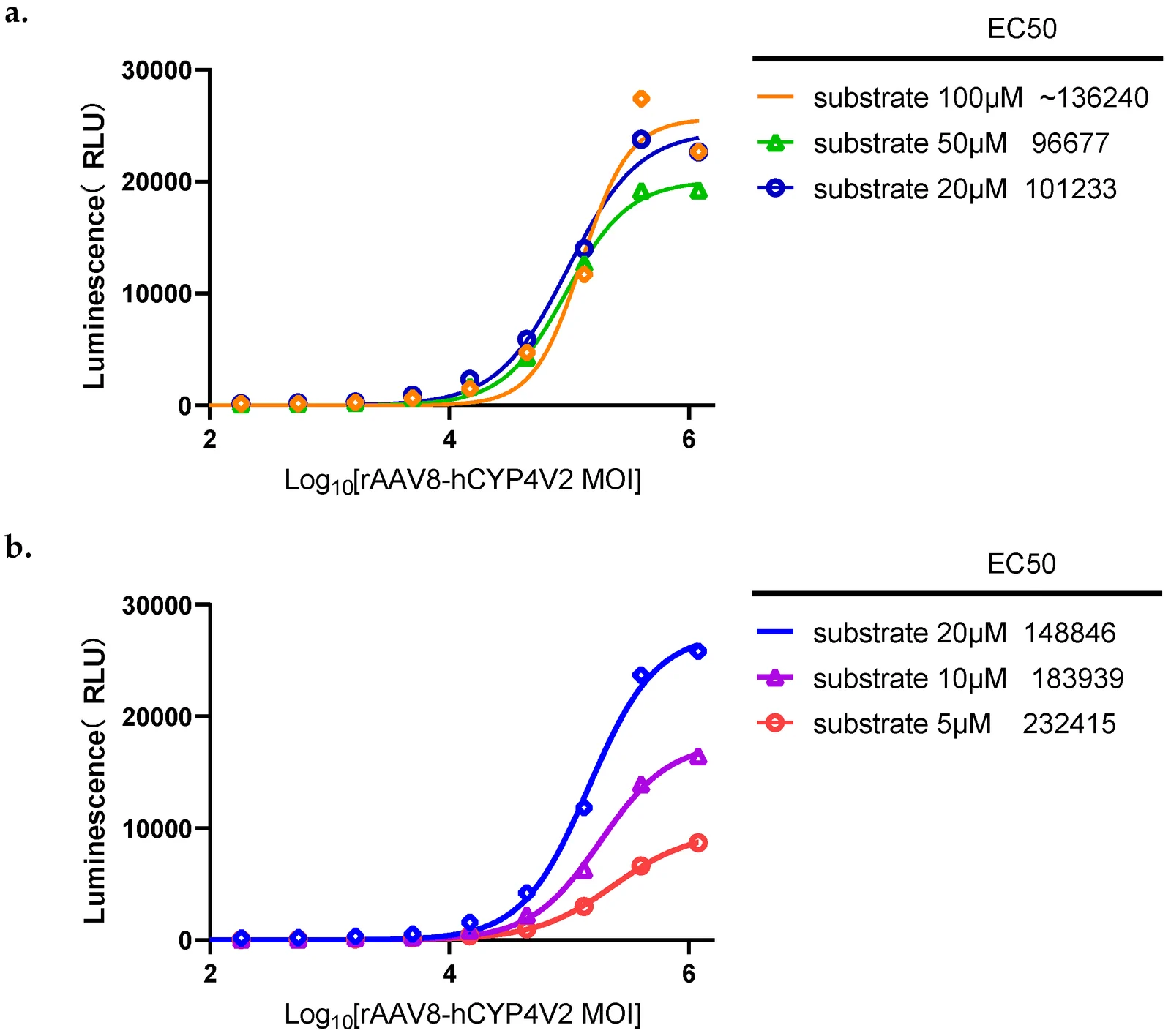
The influence of different Multi-CYP concentrations (5–100 µM) on the four-parameter fitting curve. (**a**) Four-parameter fitting curves for Multi-CYP concentrations of 100 µM, 50 µM, and 20 µM. (**b**) Four-parameter fitting curves for Multi-CYP concentrations of 20 µM, 10 µM, and 5 µM.

Using the experimental conditions determined in the previous stage, the influence of different substrate concentrations (100 µM, 50 µM, 20 µM, 10 µM, 5 µM) on the fitting results of the four parameters was further investigated. The MOI was diluted in a 3-fold series starting from 1.20 × 10^6^, with a total of 9 dilution levels ranging from 1.20 × 10^6^ to 1.83 × 10^2^. As shown in Figure 9 (and Table S10), the best four-parameter fitting results were achieved using a substrate concentration of 20 μM (at a substrate concentration of 100 μM, the four-parameter fitting results were inferior; at substrate concentrations of 50 μM, 10 μM, and 5 μM, the signal values were slightly lower). Hence, assay signal value was optimal using a substrate concentration of 20 μM, and this substrate concentration provided the best fitting results. It should be noted that the signal eventually reached saturation using this concentration. From the results, the MOI value that reached the plateau under this condition does not meet the requirements. Therefore, the next step is to reduce the cell quantity, thereby increasing the MOI, to see if the number of the plateau phase can meet the requirements.

#### 2.2.5. Validation of Plate Cell Number and Substrate Incubation Time

**Figure 10:**
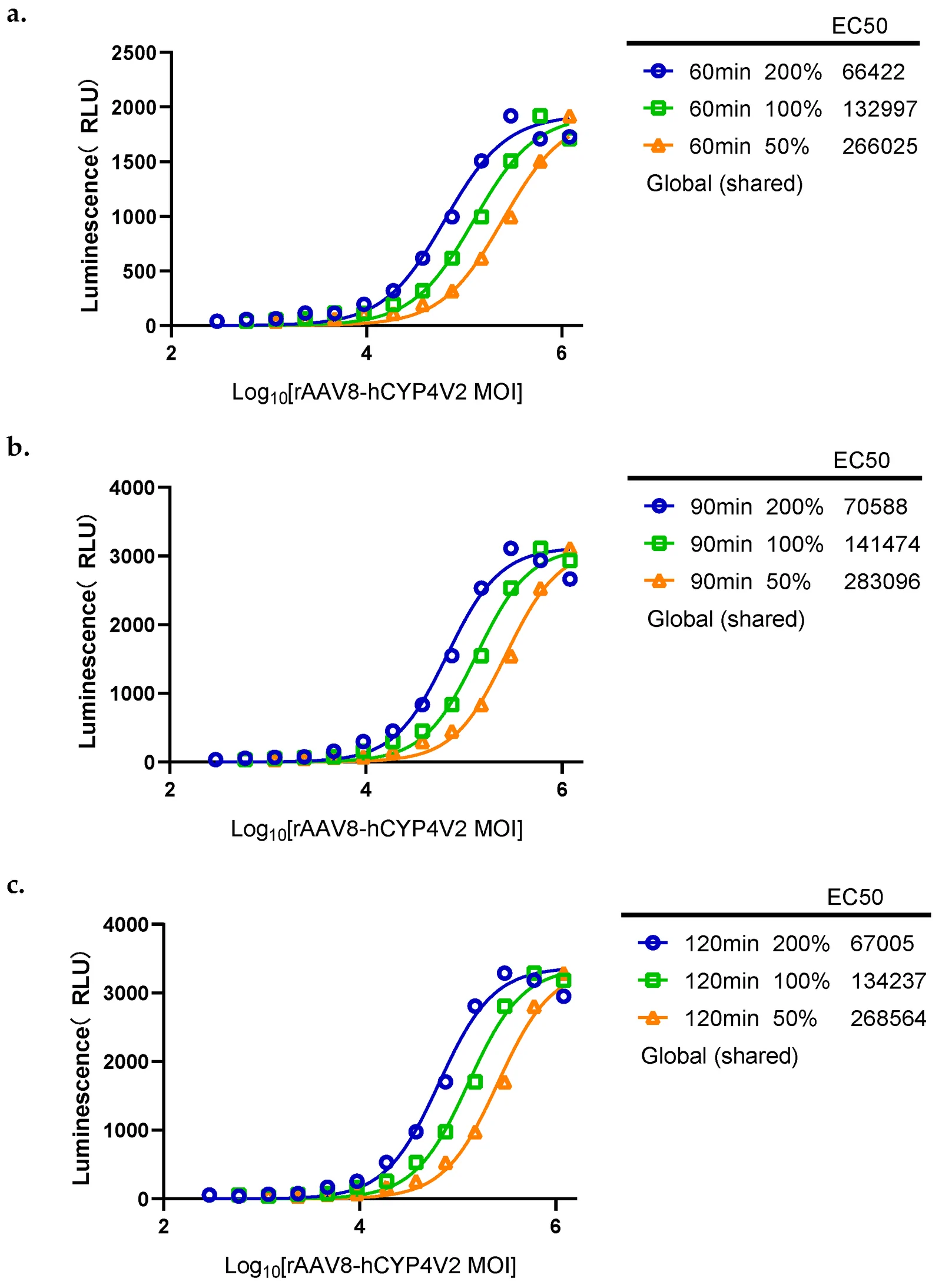
The influence of substrate incubation time (60, 90, 120 min) on relative activity. (**a**) Four-parameter fitting curves for substrate incubation at 60 min. (**b**) Four-parameter fitting curves for substrate incubation at 90 min. (**c**) Four-parameter fitting curves for substrate incubation at 120 min.

Based on the results in Section 2.2.4, the number of seeded cells was reduced from 2 × 10^4^ cells/well to 1 × 10^4^ cells/well to increase the MOI limit. The MOI was diluted in a 2-fold series starting from 2.40 × 10^6^, with a total of 12 dilution steps ranging from 2.40 × 10^6^ to 1.17 × 10^3^. The final concentration of Multi-CYP was fixed at 20 µM, and the cells were incubated in Opti-MEM medium for 60, 90, and 120 min respectively. The performance of different incubation times was evaluated by the recovery rate (RB%) of 200% and 50% samples relative to 100% samples. After cell calibration, the plateau was reached at MOIs of 1.20 × 10^6^, 6.00 × 10^5^, and 3.00 × 10^5^; the results obtained using these MOIs met all experimental requirements. At an MOI of 2.4 × 10^6^, the virus concentration was too high, and the cells were in too poor a condition to provide good data; therefore, this data point was removed during fitting. At substrate reaction times of 60 min, 90 min, and 120 min, the activities of the 200% and 50% samples (relative to the 100% sample) were 200.23% and 49.99%, 200.42% and 49.97%, and 200.34% and 49.98%, respectively (Figure 10, Table S11). The RB% values were 0.12% and −0.11%, 0.21% and −0.05%, and 0.17% and −0.03%, respectively. On the basis of these new experiments, the number of plated cells was fixed at 1.00 × 10^4^ cells per well, and the enzyme activity reaction time was fixed at 90 min ± 30 min.

### 2.3. Methods Verification and Application Results

#### 2.3.1. Exclusivity

**Figure 11:**
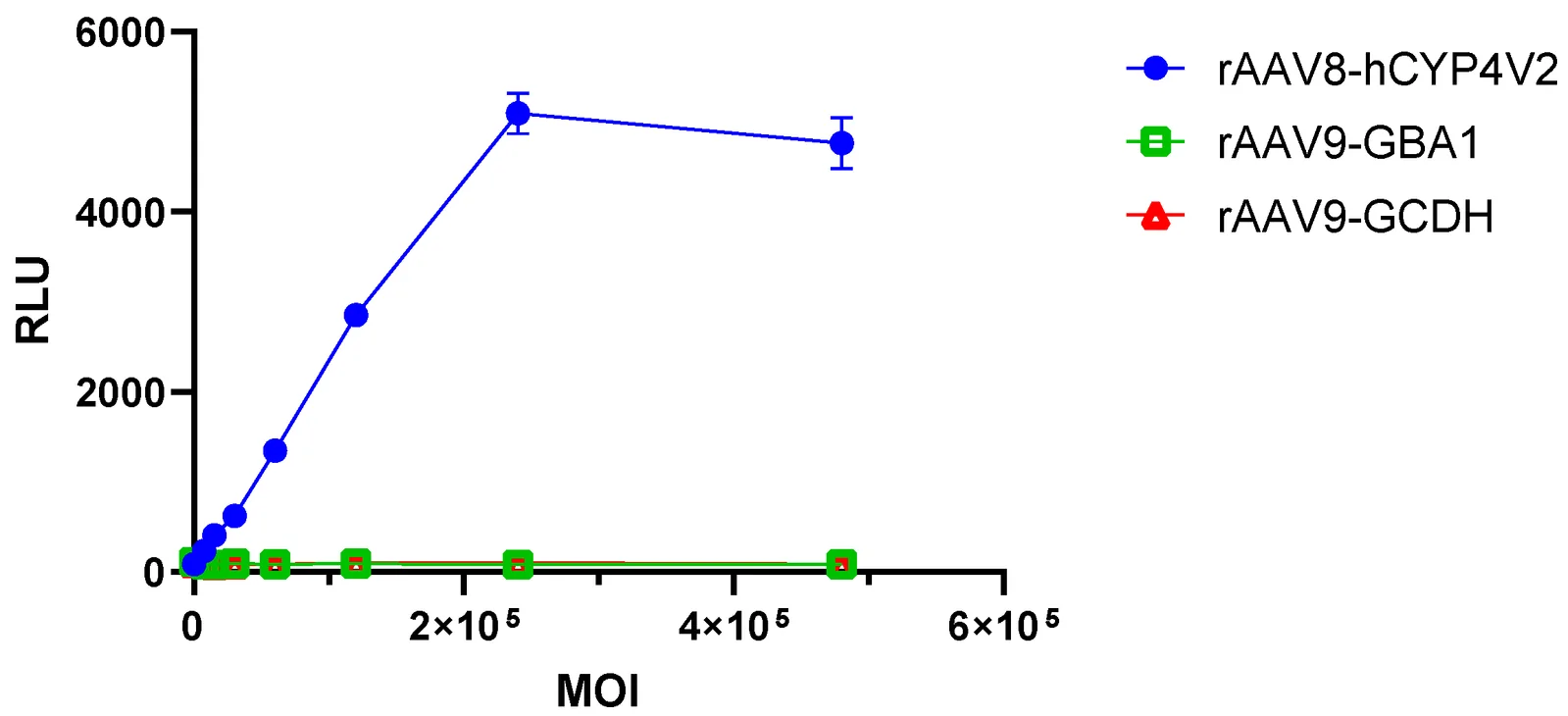
Comparison of detection signals between the target protein CYP4V2 and the non-target protein.

To verify the specificity of the method, HEK293-AAVR cells were used as the host cells. rAAV8-hCYP4V2 or two homologous non-target protein viruses (rAAV9-GBA1, rAAV9-GCDH) were respectively transduced at gradient MOIs (4.8 × 10^5^ to 7.5 × 10^3^) and the detection was carried out according to the established procedure. The detection signal values for the rAAV8-hCYP4V2 group increase with increasing MOI, reaching a peak at MOI = 2.4 × 10^5^. For the rAAV9-GBA1 and rAAV9-GCDH groups, the detection signal values do not show a significant change trend (across different MOI conditions), and the results are consistent with the negative control group (Figure 11, Table S12). Together, these results provide evidence that this method can specifically detect the activity of the CYP4V2 protein (generated by rAAV8-hCYP4V2), with no significant response to non-target proteins (generated rAAV9-GBA1 and rAAV9-GCDH groups). Hence, the developed method demonstrates good specificity.

#### 2.3.2. Accuracy

Using standard operating procedures, spiked recovery rates and relative bias (RB) were calculated at each potency level. The obtained accuracy was 100.94% ± 2.84% (RSD = 2.81%). The relative bias (RB %) values for the repeat groups were −0.64%, −0.76%, and 4.22%, respectively (Table 1).

#### 2.3.3. Precision

Using standard operating procedures, we next obtained repeatability and intermediate precision results. The GCV% from six trials was 4.21% (Table 2), and the intermediate precision RSD was 6.16% (Table 3).

#### 2.3.4. Linearity and Range

Linear regression was performed on each experimental group, and the R^2^ values were obtained. The mean ± standard deviation was 0.9902 ± 0.0061 (RSD = 0.61%, n = 9), indicating that our luminescent substrate assay exhibits good linearity within the MOI range of 1.17 × 10^3^ to 2.4 × 10^6^ (Table 4).

#### 2.3.5. Durability

**Figure 12:**
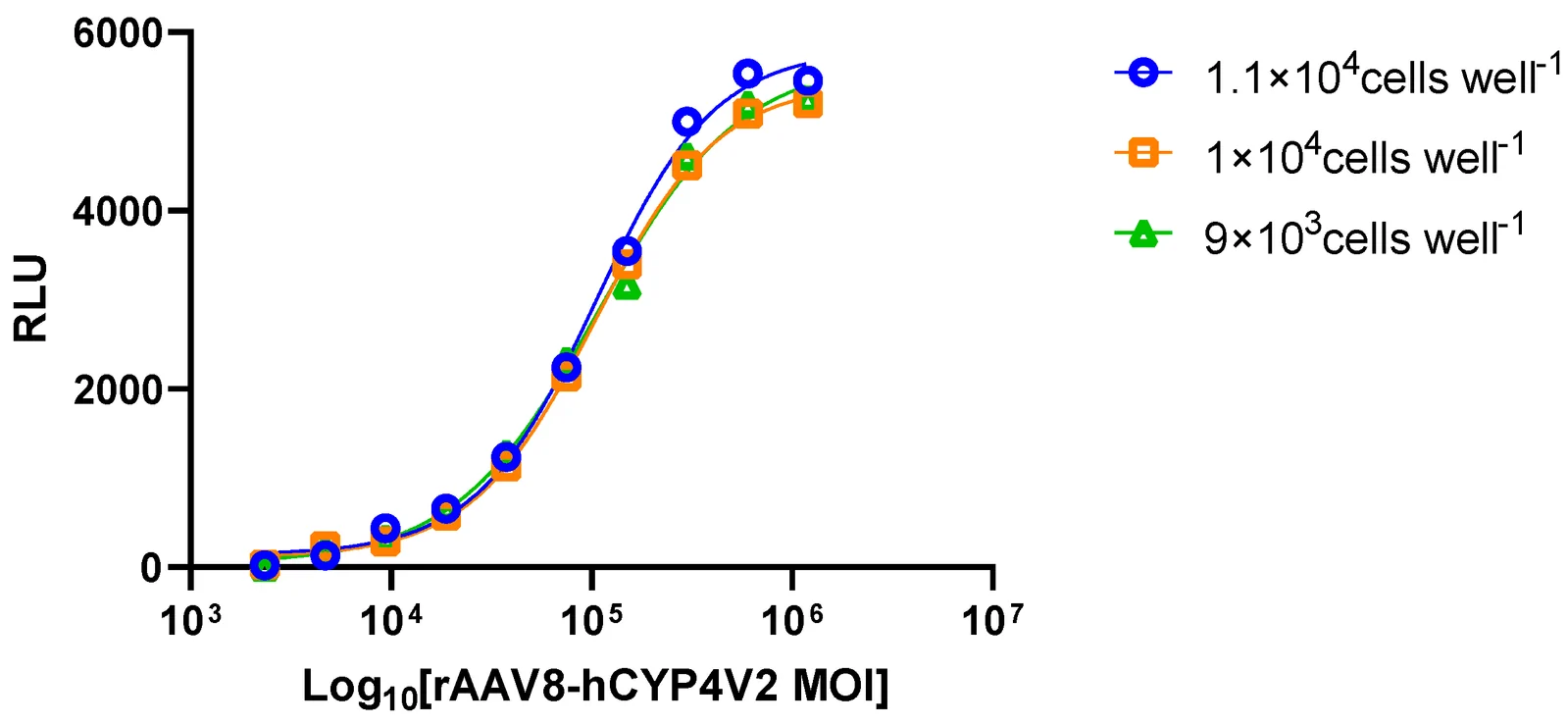
Full assay results obtained after viral transduction using different numbers of seeding cells.

After the above optimization experiments, the standard number of seeded cells was selected to be 1.00 × 10^4^ cells per well. On this basis, we next evaluated the effect of a ±10% variation in the seeding amount (1.1 × 10^4^ and 9 × 10^3^) on the measurement results to assess the method’s robustness. Cells were seeded at different numbers (1.1 × 10^4^ cells per well, 1 × 10^4^ cells per well, and 9 × 10^3^ cells per well) and CYP4V2 enzyme activity was then measured. After four-parameter fitting, the R^2^ values of the detection signals obtained were 0.996, 0.995, and 0.995, respectively. The activities relative to the standard (1 × 10^4^ cells per well) were 85.78% and 94.98% for 1.1 × 10^4^ and 9 × 10^3^ cells per well, respectively. The RB% values were −14.22% and −5.02%, respectively, and the slope ratios were 0.904 and 0.832, respectively. Together, these results show that the method has good robustness with respect to the number of seeded cells (Figure 12, Table 5).

Taking into account the accuracy and precision testing described above, the effects of HEK293-AAVR cell passage number (passage P28 vs. passage P32) on the enzyme activity assay results were evaluated next. The mean relative activity measured at passage P28 was 100.09% (RSD = 2.37%, n = 3), while the mean relative activity at passage P32 was 100.29% (RSD = 9.41%, n = 3). The intermediate precision RSD of the combined six measurements was 6.16%, indicating that cell passage number had no significant impact on the precision of this method. Hence, the assay method presented here demonstrates good robustness.

**Figure 13:**
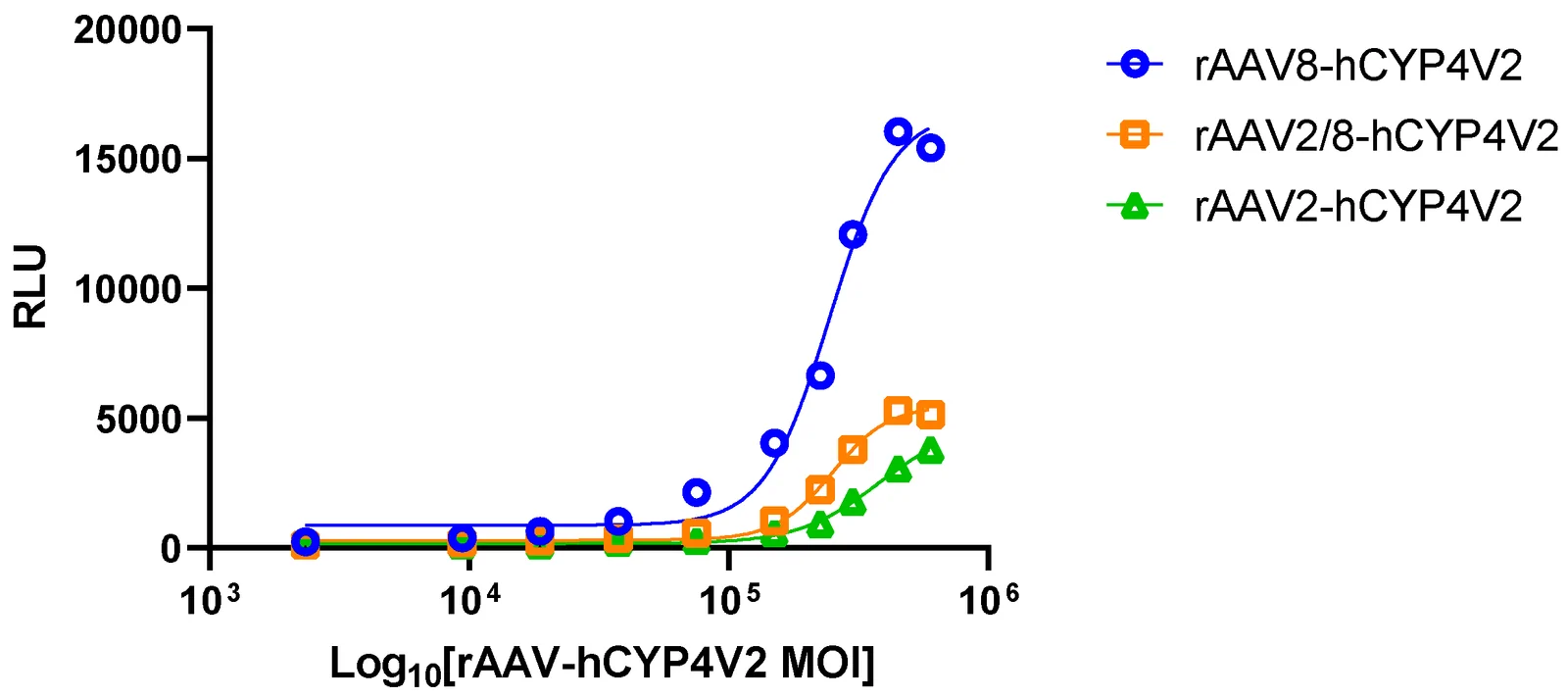
Method generalization: Comparison of enzymatic activities of three AAV serotypes (AAV8, AAV2/8, AAV2) products.

Finally, to evaluate the generalizability of this method and test whether it can effectively distinguish the activity differences among different gene therapy products, we selected three rAAV-hCYP4V2 products and assessed their CYP4V2 enzymatic activity. As shown in Figure 13 and Table S13, HEK293-AAVR cells were infected with the three rAAV-hCYP4V2 products at equal MOI. Using our luminescence assay to measure biological activity, we found that the results obtained with all three rAAV-CYP4V2 products could be fitted with four-parameter curves. However, the luminescence response values of the rAAV2/8-CYP4V2 and rAAV2-CYP4V2 products were lower than that of the rAAV8-CYP4V2 product, only reaching approximately 30% of its response value; thus, the rAAV8-CYP4V2 product demonstrated stronger CYP4V2 enzymatic activity than the other two products. Using rAAV8-hCYP4V2 as the reference standard, the slope ratios of rAAV2/8-hCYP4V2 and rAAV2-hCYP4V2 were 1.13 and 0.80, respectively, both within the predefined acceptable range (0.80 to 1.25), demonstrating good curve parallelism. Hence, the developed method was able to effectively distinguish differences in CYP4V2 enzymatic activity among products from different serotypes. Accordingly, if the reference standards for each rAAV-CYP4V2 product are used as a reference, the stated method can be used universally to determine relative biological activity.

## 3. Discussion

In this study, we successfully established and validated a CYP4V2 enzyme activity assay for quantitative assessment of the relative biological activity of rAAV-hCYP4V2 gene therapy products based on the bioluminescent substrate Multi-CYP. Furthermore, method validation results demonstrate that the new assay meets the technical requirements of the Chinese Pharmacopoeia General Chapter 9401 and ICH Q2 (R2) for biological assay methods of biologics in terms of specificity, accuracy, precision, linearity, and robustness.

Compared with LC-MS/MS-based fatty acid hydroxylase activity detection methods, the method reported here offers several clear operational advantages. The LC-MS/MS approach achieves absolute quantification of CYP4V2 enzyme activity by detecting the specific ω-hydroxylated product of the lauric acid substrate [22]. Hence, this matchless detection method accurately reports on the true biological function of CYP4V2. However, the LC-MS/MS detection method involves cumbersome sample pretreatment steps such as lipid extraction and derivatization, has a long analysis cycle per run, and requires high equipment investment and maintenance costs. In contrast, the bioluminescent substrate method described here does not require chromatographic separation or complex sample pretreatment, and the entire assay can be performed in a microplate. In a single experiment, hundreds of samples can be analyzed in parallel, greatly shortening the analysis cycle. In addition, our new method is amenable to automation. Certainly, microplate dispensing, incubations, and signal reading can all be performed using an automated liquid handling workstation and a microplate reader. Therefore, our new bioluminescent substrate method is more practical for applications such as activity screening in early-stage R&D, process condition optimization, and rapid comparisons among multiple batches of samples.

Although our new method demonstrates significant advantages in terms of throughput and operational convenience, it still has certain limitations. First, the methodology was validated in the present study using an in vitro cell transduction model, whereas clinical samples may involve more complex matrices (such as vitreous fluid, retinal tissue homogenates, etc.), and the potential interference effects of different sample matrices on the luminescent signal must be further evaluated. Second, our method uses the artificial bioluminescent substrate Luciferin-MultiCYP to measure enzyme activity; thus, it reflects the hydroxylation capability of CYP4V2 toward this specific substrate, not its ω-hydroxylation activity toward its natural fatty acid substrates (such as lauric acid, DHA). Although previous studies have shown that MultiCYP is a universal substrate for multiple CYP450 enzymes [23], the correlation and consistency between this method and LC-MS/MS in reflecting the true biological function of CYP4V2 still require experimental validation. To address this, this study used the absolute quantification value obtained by LC-MS/MS from the reference batch (P1) (150.2 pg/mL/min) as the calibration standard, converted the relative activity percentages measured by the fluorescent method into absolute activity values, and directly compared these calculated values with the actual LC-MS/MS measurements across batches. The results (Table 6) showed that the recalculated value for batch P2 (153.2 pg/mL/min) deviated from the LC-MS/MS measurement (144.6 pg/mL/min) by only 5.9%, indicating good agreement between the two methods for this batch. However, the recalculated value for batch P3 (162.3 pg/mL/min) was significantly higher than the measured value (98.9 pg/mL/min), resulting in a deviation of 64.1%. This significant difference is mainly attributed to the overly complex operation of the LC-MS/MS method during the detection process, which leads to variations in protein extraction efficiency among different batches, thereby increasing the overall experimental variability and ultimately resulting in deviations in the quantitative results between the two methods. Finally, our method provides relative biological activity (a potency ratio based on a reference standard), rather than the absolute catalytic rate of the enzyme (that is, the amount of product generated per unit time); therefore, when comparing absolute activity across batches, standardization with a reference standard is required.

In summary, the bioluminescent substrate method established in this study demonstrates strong specificity, high accuracy, good precision, and several advantages, including ease of operation and high throughput. Moreover, it can effectively support process development and quality control Important directions of future research will include the migration and adaptation of the bioluminescent substrate method to a high-throughput automated analysis platform, and validation of a correlation with the LC-MS/MS method.

## 4. Materials and Methods

### 4.1. Main Instruments

Cell counting, passaging, and culture were performed using a cell counter (Thermo, Waltham, MA, USA) Countess II FL, a Class II biosafety cabinet (Suzhou Antai Air Technology Co., Ltd., Suzhou, China) BSC-1304 II A2, a CO_2_ incubator (Thermo) VIOS I60i, a refrigerated high speed centrifuge (Merrick Instruments Co., Ltd., Shanghai, China) MH1650, and a medical centrifuge (Hunan Xiangyi Laboratory Instrument Development Co., Ltd., Xiangtan, China) 7DZ4-WS. Bioluminescent signal intensity was measured using a microplate reader (Molecular Devices (Shanghai) Co., Ltd., Shanghai, China) i3X.

### 4.2. Critical Reagents

Multi-CYP, 2FEME, BE, PFBE, and CEE were purchased from Medicilon (Shanghai Medicilon Inc., Shanghai, China). Hydroxyurea and DMSO were purchased from Sigma (Sigma-Aldrich, St. Louis, MO, USA). The P450-Glo™ Screening System and Luciferin Detection Reagent with esterase, as well as the CellTiter-Glo^®^ Luminescent Cell Viability Assay, were purchased from Promega (Promega Corporation, Madison, WI, USA). Opti-MEM I (1×), DMEM basic (1×), DMEM (1×) + GlutaMAX-I, TrypLE™ Express, FBS, and PBS pH 7.4 (1×) were all purchased from Gibco (Thermo Fisher Scientific, Waltham, MA, USA).

### 4.3. Virus

The rAAV2-hCYP4V2, rAAV8-hCYP4V2, and rAAV2/8-hCYP4V2 viral samples were obtained from archived samples held by the National Institutes for Drug Control in China.

### 4.4. Methodology

CYP4V2 protein belongs to the cytochrome P450 protein family 4 and demonstrates hydroxylase activity [1]. CYP4V2 is able to convert a luciferin precursor derivative, Luciferin-MultiCYP(methyl(*S*)-2-(6-methoxybenzo[d]thiazol-2-yl)-4,5-dihydrothiazole-4-carboxylate, Cas number: 916661-67-1)) into a luciferin methyl ester precursor; in the presence of esterase, this fluorescent precursor of methyl fluorescein generates D-fluorescein, and D-fluorescein reacts with firefly luciferase to produce a biological luminescence signal.; the intensity of this signal is directly proportional to the enzymatic activity of the CYP4V2 protein [18].

#### 4.4.1. Cell Culture and Transduction

HEK293-AAVR cells were seeded into 96-well plates at a density of 1 × 10^4^ cells per well and incubated overnight at 37 ± 2 °C. The cells were transduced with rAAV-hCYP4V2 at an MOI of 1.17 × 10^3^ to 2.4 × 10^6^ and incubated in a 37 °C CO_2_ incubator for 72 h ± 2 h. Cell culture supernatant was used as the reaction medium.

#### 4.4.2. Enzyme Activity Luminescence Detection

Dissolve the Multi-CYP substrate (Luciferin-MultiCYP, MedChemExpress) in Opti-MEM medium to prepare a reaction substrate working solution with a final concentration of 20 µM. Remove the culture supernatant of the transduced cells (plating density of 1 × 10^4^ cells per well, virus incubation for 72 h) and add 50 µL of the reaction substrate working solution to each well. Incubate at 37 °C in a 5% CO_2_ incubator for 90 min ± 30 min (i.e., substrate incubation time). After incubation, transfer 25 µL of the cell supernatant to a new 96-well plate, add 50 µL of the luciferase detection reagent containing esterase (Luciferin Detection Reagent with esterase, Promega, V8931), and incubate at 37 °C in the dark for 15 min ± 5 min. Immediately measure the bioluminescence signal (relative light units, RLU) using an enzyme detector.

#### 4.4.3. Enzyme Activity Calculation

Four-parameter logistic regression (4PL) model fitting was performed on the concentration effect data of the reference and test samples using SoftMax Pro 7.0 software. To fit the S-shaped dose response curve, the log value of MOI (range, 1.17 × 10^3^–2.40 × 10^6^) was taken as the horizontal axis (X) and the mean luminescent intensity as the vertical axis (Y).

The four-parameter regression equation was as follows:

Y = (A − D)/[1 + (X/C)B] + D

Here, A is the upper asymptote (maximum response value), D is the lower asymptote (minimum response value), C is the half-maximal effective concentration (EC50), and B is the slope factor.

The relative activity of the test samples was calculated using the following formula:

Relative Activity (%) = (Reference EC_50_/Test Sample EC_50_) × 100%.

To clarify, the higher the relative activity value, the lower the MOI required for the test sample to achieve half of the maximum effect, indicating a higher relative transduction/expression efficiency. The validity of each single detection result was determined using the “Experimental Results Interpretation” generated by the SoftMax Pro software. The relative biological activity of each sample and its 95% confidence interval are reported as “Relative Potency” and “Confidence Interval”.

#### 4.4.4. Statistical Analysis

All validation experiments were conducted independently at least three times (n ≥ 3), and the data are presented as mean ± standard deviation (SD). Precision was evaluated by relative standard deviation (RSD), where RSD (%) = (SD/mean) × 100%. In the time-course experiments, the linear relationship between reaction time and product concentration was assessed by linear regression analysis (F test); a difference was considered statistically significant at *p* < 0.05, indicating that the linear relationship holds. Statistical analyses and figure plotting were performed using GraphPad Prism 10.1.2.

### 4.5. Methodology Validation

#### 4.5.1. Exclusivity

The specificity of the method was verified by separately transducing cells with an MOI gradient of viruses carrying the target protein CYP4V2 or one of two different homologous nontarget proteins. Bioluminescence was subsequently detected after substrate incubation.

#### 4.5.2. Accuracy

Accuracy was taken as the relative bias (RB %) between the sample’s actual relative activity % and the theoretical relative activity %.

#### 4.5.3. Precision

Repeatability and Intermediate precision are reported using GCV % and the relative standard deviation (RSD) between groups, respectively. Repeatability was assessed using six independent replicate measurements of the same batch of samples within a specified time by the same operator, following the established standard operating procedures; the enzyme activity values obtained from the six measurements were then used for the calculation. Repeatability was then evaluated using the geometric coefficient of variation (GCV %). The standard deviation of the relative activity was calculated after logarithmic transformation and, according to the formula, GCV % = (10^SDlog^ − 1) × 100%, where SDlog is the standard deviation after taking the common logarithm of the relative activity. Intermediate precision was assessed using two different operators measuring the enzyme activity of virus samples on two different working days, and it is characterized using relative standard deviation (RSD).

#### 4.5.4. Linearity and Range

All measurements were performed using standard operating procedures and the sample data were processed using a four-parameter regression model. To determine the half-maximal effective concentration (EC_50_), sample MOI was taken as the horizontal coordinate and mean luminescence intensity as the vertical coordinate. The procedure used three repeats to assess linearity; the range was determined during methodological parameter optimization.

#### 4.5.5. Durability

To evaluate durability, we assessed the effects of different generations of cells and different cell seeding numbers on the results of enzyme activity assays. In addition, we assessed the applicability of the new method to different rAAV-hCYP4V2 gene therapy products.

## Figures and Tables

**Table 1:** Accuracy Results.

Repetitive Group	Theoretical Activity	Relative Activity	Accuracy	Relative Bias (RB %)
1	50%	49.68%	99.36%	−0.64%
2	100%	99.24%	99.24%	−0.76%
3	200%	208.44%	104.22%	4.22%

Note: Relative activity = (measured EC_50_/theoretical EC_50_) × 100%; RB % = (relative activity − theoretical activity)/theoretical activity × 100%.

**Table 2:** Repeatability results.

Repetitive Group	Theoretical Activity	Relative Activity	GCV %
1	100%	102.3%	4.21%
2		97.56%	
3		100.42%	
4		94.88%	
5		103.08%	
6		106.52%	

Note: GCV % = Geometric Variance Coefficient.

**Table 3:** Intermediate Precision Results.

Repetitive Group	Theoretical Activity	Relative Activity	RSD
1 (Operator A)	100%	102.30%	6.16%
2 (Operator A)		97.56%	
3 (Operator A)		100.42%	
4 (Operator B)		110.00%	
5 (Operator B)		99.75%	
6 (Operator B)		91.12%	

Note: Operator A’s data are the results of three repeated measurements identical to those in Table Operator B’s data are the results of three independent measurements from another working day; Operator A used cells at passage P28, and Operator B used cells at passage P32. RSD = Relative Standard Deviation.

**Table 4:** Linearity analysis results.

Repetitive Group	R^2^
1	0.991
2	0.990
3	1.000
4	0.991
5	0.985
6	0.997
7	0.980
8	0.986
9	0.992

**Table 5:** Full assay results showing plating cell quantity durability.

Cell Plate Number	Relative Activity	R^2^	Slope Ratio
1.1 × 10^4^ cells/well	85.78%	0.996	0.904
9 × 10^3^ cells/well	94.98%	0.995	0.832
1 × 10^4^ cells/well	1.00	0.995	1.00

Note: The slope ratio was computed based on 1 × 10^4^ cells per well.

**Table 6:** Cross-method comparison of fluorescence-based back-calculated activity versus LC-MS/MS absolute quantification (normalized to reference batch P1).

Batch	Fluorescence Relative (%)	Back-Calculated Absolute by Fluorescence (pg/mL/min)	LC-MS/MS Absolute (pg/mL/min)	Deviation
P1 (reference)	100	150.2	150.2	-
P2	102	153.2	144.6	5.9%
P3	108	162.3	98.9	64.1%

Note: Fluorescence values were normalized to P1 = 100% (correction factor: 100/99). Back-calculated absolute = normalized fluorescence % × P1 LC-MS/MS (150.2 pg/mL/min). Deviation (%) = (back-calculated − LC-MS/MS)/LC-MS/MS × 100%. LC-MS/MS is the reference method.

## Data Availability

The data presented in this study are available upon reasonable request from the first author.

## Supplementary Materials

The following supporting information can be downloaded at: https://www.mdpi.com/article/10.3390/molecules31162811/s1.

## Abbreviations

The following abbreviations are used in this manuscript:

AAV	Adeno-associated virus
BCD	Bietti crystalline dystrophy
DHA	docosahexaenoic acid
EPA	eicosapentaenoic acid
CYP4V2	Cytochrome P450 family 4 subfamily V member 2
DMEM	Dulbecco’s modified Eagle medium
Opti-MEM	Opti-MEM™ I Reduced-Serum Medium
HU	Hydroxyurea
MOI	Multiplicity of infection
ICH	International Council for Harmonisation of Technical Requirements for Pharmaceuticals for Human Use
PBS	Phosphate-buffered saline
CO_2_	Carbon Dioxide
4PL	Four-parameter logistic regression
GCV	Geometric Coefficient of Variation
R^2^	Coefficient of determination
rAAV-hCYP4V2	Recombinant adeno-associated virus carrying the human CYP4V2 gene
RSD	Relative standard deviation
SD	Standard deviation
