# Supplementary material for: Development and Validation of a Method to Determine CYP4V2 Enzyme Activity in rAAV-hCYP4V2 Gene Therapy Products Using a Bioluminescent Substrate Assay
Source: Molecules. 2026 Aug 12;31(16):2811. doi: 10.3390/molecules31162811 (PMC13515460; doi:10.3390/molecules31162811)
Supplement: Supplementary file 1 [file molecules-31-02811-s001.zip › molecules-4470363-supplementary.pdf]

Table S1. Detection results of six substrates under the condition of fluid replacement 24 hours after transduction (comparison between supernatant and lysate)

substrate type	cell supernatant		cell lysate		cell supernatant	cell lysate
	rAAV8-hCYP4V2(+)	rAAV8-hCYP4V2(-)	rAAV8-hCYP4V2(+)	rAAV8-hCYP4V2(-)	difference	difference
BE	34	21	0	8	13	-8
PFBE	50	44	3	29	6	-26
2FEME	2026	2002	81	50	24	31
CEE	34	52	8	8	-18	0
ME-EGE	120	110	24	18	10	6
Multi-CYP	199	463	13	24	-264	-11

Note：The data in the table represent a single representative experiment. The values are the luminescence signal values (RLU), and "difference" = rAAV8-hCYP4V2(+) - rAAV8-hCYP4V2(-).

Table S2. Detection results of six substrates under the condition of fluid replacement 72 hours after transduction (comparison between supernatant and lysate)

substrate type	cell supernatant		cell lysate		cell supernatant	cell lysate
	rAAV8-hCYP4V2(+)	rAAV8-hCYP4V2(-)	rAAV8-hCYP4V2(+)	rAAV8-hCYP4V2(-)	difference	difference
BE	21	29	8	3	-8	5
PFBE	47	50	5	13	-3	-8
2FEME	1992	1391	78	44	601	34
CEE	71	55	13	16	16	-3
ME-EGE	84	68	21	18	16	3
Multi-CYP	1310	706	71	55	604	16

Note：The data in the table represent a single representative experiment. The values are the luminescence signal values (RLU), and "difference" = rAAV8-hCYP4V2(+) - rAAV8-hCYP4V2(-).

Table S3. Detection results of different Multi-CYP concentrations under the condition of fluid replacement 24 hours after transduction (comparison between supernatant and lysate)

substrate concentration	type of culture medium	cell supernatant		cell lysate		cell supernatant	cell lysate
		rAAV8-hCYP4V2(+)	rAAV8-hCYP4V2(-)	rAAV8-hCYP4V2(+)	rAAV8-hCYP4V2(-)	difference value	difference value
Multi-CYP-100μM	Opti-MEM (Serum free)	580	277	170	68	303	102
Multi-CYP-10μM		1469	1218	110	42	251	68
Multi-CYP-1μM		1106	520	29	10	586	19
Multi-CYP-0.1μM		146	152	3	8	-6	-5
Multi-CYP-100μM	Complete medium	76	47	39	13	29	26
Multi-CYP-10μM		186	141	13	13	45	0
Multi-CYP-1μM		246	254	16	5	-8	11
Multi-CYP-0.1μM		115	133	3	13	-18	-10

Note：The data in the table represent a single representative experiment. The values are the luminescence signal values (RLU), and "difference" = rAAV8-hCYP4V2(+) - rAAV8-hCYP4V2(-).

Table S4. Detection results of different Multi-CYP concentrations under the condition of fluid replacement at 72 hours after transduction (comparison between supernatant and lysate)

substrate concentration	type of culture medium	cell supernatant		cell lysate		cell supernatant	cell lysate
		rAAV8 -hCYP4 V2(+)	rAAV8 -hCYP4 V2(-)	rAAV8 -hCYP4 V2(+)	rAAV8 -hCYP4 V2(-)	difference value	difference value
Multi-CYP-100μM	Opti-MEM(Serum free)	1310	230	371	58	1080	313
		1843	719	94	26	1124	68
Multi-CYP-10μM							
Multi-CYP-1μM		708	523	3	29	185	-26
Multi-CYP-0.1μM		193	81	10	8	112	2

Note: The data in the table represent a single representative experiment. The values are the luminescence signal values (RLU), and "difference" = rAAV8-hCYP4V2(+) - rAAV8-hCYP4V2(-).

Table S5. Comparison results of viral transduction efficiency between HEK293 and HEK293-AAVR cell lines

substrate concentration	type of culture medium	HEK293		HEK293-AAVR	
		rAAV8-hCYP4 V2(+)	rAAV8-hCYP4 V2(-)	rAAV8-hCYP4 V2(+)	rAAV8-hCYP4 V2(-)
Multi-CYP-10μM	Opti-MEM (Serum free)	570	282	2962	225
Multi-CYP-50μM		625	397	5440	389
Multi-CYP-100μM		536	455	6608	557

Note: The data in the table represent a single representative experiment. The numerical values are the luminescence signal values (RLU).

Table S6. The detection window is the difference in RLU values between rAAV8-hCYP4V2(+) and rAAV8-hCYP4V2(-).

**substrate concentration**	**type of culture medium**	**MOI=4.8E5**		**MOI=1E5**		**MOI=4.8E5**	**MOI=1E5**	**MOI= 4.8E5**	**MOI =1E5**
		**rAAV8-hCYP4V2(+)**	**rAAV8-hCYP4V2(-)**	**rAAV8-hCYP4V2(+)**	**rAAV8-hCYP4V2(-)**	**difference**	**difference**	**ratio**	**ratio**
Multi-CYP-100μM	Opti-MEM(Serum free)	6608	557	1388	329	6051	1059	11.9	4.2
Multi-CYP-50μM		5440	389	1155	290	5051	865	14.0	4.0
Multi-CYP-10μM		2962	225	549	159	2737	390	13.2	3.5
Multi-CYP-100μM	complete medium	2355	337	795	207	2018	588	7.0	3.8
Multi-CYP-50μM		2180	186	706	125	1994	581	11.7	5.6
Multi-CYP--10μM		3328	128	630	63	3200	567	26.0	10.0
2FEME-100μM	Opti-MEM(Serum free)	25939	20191	21173	20690	5748	483	1.3	1.0
2FEME-50μM		12043	9951	10738	10307	2092	431	1.2	1.0
2FEME-10μM		2483	2360	2256	2457	123	-201	1.1	0.9
2FEME-100μM	complete medium	30056	11888	15846	11143	18168	4703	2.5	1.4
2FEME-50μM		14215	8655	9376	8966	5560	410	1.6	1.0
2FEME-10μM		3079	2481	2329	2460	598	-131	1.2	0.9

Note:The values in the table represent the luminescence signal values (RLU). "Difference" = rAAV8-hCYP4V2(+) - rAAV8-hCYP4V2(-); "Ratio" = rAAV8-hCYP4V2(+) / rAAV8-hCYP4V2(-).

Table S7. Linear regression analysis of incubation time of the substrate and detection signal under two preferred conditions

substrate concentration	type of culture medium	incubating time （h）	rAAV8 -hCYP4V2(+)	rAAV8 -hCYP4V2(-)	linear equation	R^2
Multi-CYP -100μM	Opti-MEM (Serum free)	0	81	31	Y=1363*X -109.5	0.9836
		1	1074	44		
		2	2402	71		
		3	4180	102		
Multi-CYP -10μM	complete medium	0	10	8	Y=511.2*X -180.8	0.8221
		1	240	24		
		2	450	26		
		3	1644	105		

Note:The linear regression equation (Y = a + bX) and the coefficient of determination (R^2) in the table were derived based on the measured RLU values of the rAAV8-hCYP4V2(+) group at each time point through the least squares method. Here, Y represents the chemiluminescence signal (RLU), and X represents the substrate incubation time (h).

Table S8. The test results after incubation of the substrate for 3 hours and 18 hours

	MOI=4.8×10^5		MOI=1×10^5	
	rAAV8 -hCYP4V2(+)	rAAV8 -hCYP4V2(-)	rAAV8 -hCYP4V2(+)	rAAV8 -hCYP4V2(-)
3h	6608	557	1388	329
18h	3168	494	1046	429

Table S9. The incubation time of the substrate under different MOI conditions (0 - 180 minutes) and the detection results of the detection signal

Time（min）/MOI	1.2×10^5	RSD %	2×10^5	RSD %	1×10^5	RSD %	5×10^4	RSD %
0	23.33	19.8	10.00	30.7	20.67	32.4	23.00	0
45	2206.33	2.2	2584.33	3.9	1462.67	9.8	799.00	12.1
90	2378.00	4.0	3136.67	9.3	1734.00	5.0	827.67	5.2
135	6523.33	4.0	8223.67	0.5	4428.67	3.8	2153.00	1.1
180	4535.00	0.9	5987.33	2.3	3271.00	6.0	1507.33	1.1

Note：The values in the table represent the luminescence signal values (RLU).The "average" in the table is the arithmetic mean of three replicate samples within each group.At Time = 0 min, negative values were detected in some individual double-hole tests under certain MOI conditions (possibly due to background subtraction of the instrument). These negative signal points were excluded. The mean and RSD were calculated based on the remaining valid double-holes. Additionally, the signal value at this time point was close to the detection limit and had a high RSD. Such data is for reference only and will not be included in the subsequent trend analysis.

Table S10. The four-parameter fitting results under different Multi-CYP concentrations

MOI	MOI Log10	Plate 1			Plate 2		
		Substrate 100uM	Substrate 50uM	Substrate 20uM	Substrate 20uM	Substrate 10uM	Substrate 5uM
1.20×10^6	6.079	22682	19229	22653	25829	16463	8707
4.00×10^5	5.602	27439	19195	23795	23706	13959	6626
1.33×10^5	5.125	11700	12662	14008	11857	6279	3006
4.44×10^4	4.6478	4734	4287	5913	4219	2264	993
1.48×10^4	4.1707	1487	1694	2329	1589	836	389
4.94×10^4	3.694	646	687	910	541	280	196
1.65×10^3	3.216	251	269	303	332	196	86
5.49×10^2	2.739	159	178	238	204	94	55
1.83×10^2	2.262	159	128	173	183	73	39
0	NA	144	141	139	162	105	84

Note：The units of the signal values in the table are RLU; R^2 represents the coefficient of determination; and EC50 denotes the half-maximal effect concentration.

Table S11. The influence of substrate incubation time (60, 90, 120 minutes) on relative activity results

**MOI**	**MOI Log10**	**60min**			**90min**			**120min**		
		**200%**	**100%**	**50%**	**200%**	**100%**	**50%**	**200%**	**100%**	**50%**
1.20×10^6	6.079	1727.312	1709.812	1918.852	2665.769	2936.208	3112.539	2952.7177	3185.7807	3288.2253
6.00×10^5	5.778	1709.812	1918.852	1507.703	2936.208	3112.539	2531.076	3185.7807	3288.2253	2809.0598
3.00×10^5	5.477	1918.852	1507.703	993.8596	3112.539	2531.076	1547.316	3288.2253	2809.0598	1706.0606
1.50×10^5	5.176	1507.703	993.8596	617.32	2531.076	1547.316	833.485	2809.0598	1706.0606	977.67834
7.50×10^4	4.875	993.8596	617.32	320.1867	1547.316	833.485	451.7743	1706.0606	977.67834	532.64292
3.75×10^4	4.574	617.32	320.1867	194.1612	833.485	451.7743	299.818	977.67834	532.64292	257.08294
1.88×10^4	4.273	320.1867	194.1612	112.7057	451.7743	299.818	156.9609	532.64292	257.08294	170.68358
9.38×10^3	3.972	194.1612	112.7057	115.2672	299.818	156.9609	73.70337	257.08294	170.68358	79.159566
4.69×10^3	3.671	112.7057	115.2672	63.01275	156.9609	73.70337	67.7889	170.68358	79.159566	69.265741
2.34×10^3	3.370	115.2672	63.01275	57.37746	73.70337	67.7889	55.95996	79.159566	69.265741	41.064615
1.17E×10^3	3.069	63.01275	57.37746	39.9593	67.7889	55.95996	35.48681	69.265741	41.064615	57.319705
5.86×10^2	2.768	57.37746	39.9593	/	55.95996	35.48681	/	41.064615	57.319705	/
2.93×10^2	2.467	39.9593	/	/	35.48681	/	/	57.319705	/	/
R^2		0.9878	0.9891	0.99	0.9851	0.9928	0.991	0.983	0.9944	0.993
EC50		6.64E×10^4	1.33×10^5	2.66×10^5	7.06×10^4	1.41×10^5	2.83×10^5	6.70×10^4	1.34×10^5	2.69×10^5
relative activity%		200.23%	/	49.99%	200.42%	/	49.97%	200.34%	/	49.98%

RB%	0.12%	/	-0.01%	0.21%	/	-0.05%	0.17%	/	-0.03%

Note："/" indicates that under this MOI condition the signal value is below the detection limit or was not included in the four-parameter regression analysis. RB% = [(measured relative activity - theoretical relative activity) / theoretical relative activity] × 100%.

Table S12. Exclusive outcome

MOI	Repeat	rAAV8-hCYP4V2 signal value	CV%	rAAV9-GBA1 signal value	CV%	rAAV9-GCDH signal value	CV%
4.8×10^5	1	4507	5.9	97	7.1	102	20.2
	2	5061		86		94	
	3	4713		86		68	
2.4×10^5	1	5333	4.5	86	29.5	125	18.2
	2	5055		102		97	
	3	4883		55		89	
1.2×10^5	1	2857	0.5	81	14.0	81	17.3
	2	2862		105		115	
	3	2834		86		102	
6.0×10^4	1	1427	5.8	81	9.0	86	23.9
	2	1341		89		68	
	3	1270		97		110	
3.0×10^4	1	546	12.2	125	32.9	91	11.9
	2	698		71		78	
	3	622		76		99	
1.5×10^4	1	392	10.2	71	20.1	68	27.2
	2	455		76		60	
	3	376		102		99	
6	1	209	14.7	99	22.9	102	6.5
	2	269		63		102	
	3	212		94		91	
0	1	81	13.2	107	5.7	107	16.2
	2	78		107		78	
	3	99		118		102	

Note: The unit of the signal values in the table is RLU, and CV% represents the coefficient of variation of the three replicate wells under the same MOI condition.

Table S13. Full assay results demonstrating Methodology Generalizability

MOI	rAAV8-hCYP4 V2	RSD %	rAAV2/8-hCYP4 V2	RSD %	rAAV2-hCYP4 V2	RSD %
600000.000	15404.500	0.1	5138.500	10.1	3800.500	2.0
450000.000	16043.500	8.1	5288.000	1.2	3074.000	3.0
300000.000	12069.500	2.5	3790.500	7.7	1802.000	14.6
225000.000	6636.000	7.0	2234.000	1.4	944.000	15.0
150000.000	4037.500	2.6	1041.500	5.5	586.500	10.7
75000.000	2138.500	11.1	574.500	0.6	285.000	24.8
37500.000	1025.000	10.1	356.000	6.4	209.000	8.1
18750.000	642.500	10.7	256.500	1.4	127.000	3.3
9375.000	396.500	10.5	185.000	9.2	121.500	9.9
2343.750	234.500	14.8	108.000	3.9	117.500	11.4

Note：The values in the table represent the luminescence signal values (RLU). The "average" in the table is the arithmetic mean of three replicate samples within each group.
